# Controlled Assembly of Fluorophores inside a Nanoliposome

**DOI:** 10.3390/molecules28020911

**Published:** 2023-01-16

**Authors:** Hiroaki Konishi, Eiji Nakata, Futa Komatsubara, Takashi Morii

**Affiliations:** Institute of Advanced Energy, Kyoto University, Uji, Kyoto 611-011, Japan

**Keywords:** DNA nanostructure, DNA origami, nanoliposome, compartment, encapsulation

## Abstract

Cellular compartmentalization plays an essential role in organizing the complex and multiple biochemical reactions in the cell. An artificial compartment would provide powerful strategies to develop new biochemical tools for material production and diagnosis, but it is still a great challenge to synthesize the compartments that encapsulate materials of interest while controlling their accurate locations, numbers, and stoichiometry. In this study, we evaluated chemical characteristics of a liposome-encapsulated compartment, which has great potential to locate various materials of interest with precise control of their locations and numbers in the compartment. A nanoliposome was constructed inside a ring-shaped DNA origami skeleton according to the method of Yang et al., and further equipped with a double-stranded DNA platform to assemble molecules of interest in the nanoliposome. Upon formation of the nanoliposome, a pH-sensitive fluorophore on the bridged platform showed little or no response to the pH change of the outer buffer, ensuring that the molecules assembled on the platform are effectively shielded from the outer environment. The ring-shaped DNA skeleton equipped with a double-stranded DNA platform allows spatial assembly of several functional molecules inside the nanoliposome to isolate them from the outer environment.

## 1. Introduction

Numerous chemical reactions proceed in parallel with high efficiency to drive cellular metabolism in living cells. This is accomplished by the complex but well-organized spatial arrangement of enzymes, which often relies on the specific scaffolds of proteins or membranes to achieve the high efficiency and the specificity of the sequential chemical reactions. In some cases, enzymes are further arranged in the isolated space of compartment [1]. For example, ribulose 1,5-bisphosphate carboxylase/oxygenase (RuBisCO) and carbonic anhydrase are packed inside a proteinaceous compartment of carboxysome, which is believed to increase the reaction rate of RuBisCO by increasing the concentration of the substrate [2]. A lipid membrane defines compartments that play important roles in many biological processes in cells. Enzymes and substrates encapsulated inside the compartment are separated from the outer environment, thus the flux of substrates and their reactions are finely regulated [3].

Inspired by the cellular compartments, artificial compartments have been synthesized by using various materials, such as proteins [4], lipids [5], and synthetic polymers [6,7]. However, application of these materials faces limitations due to the difficulty in controlling the accurate locations, numbers, and stoichiometry of the material of interest, such as dyes and enzymes [8]. DNA nanostructures have attracted great interest for their structural programmability and accurate addressability in applying as scaffolds to spatially arrange the molecules of interest [9]. Various materials, such as dyes [10,11], nano particles [12], nucleic acids [13], and proteins [14,15], have been assembled on the DNA scaffold to execute unique functions depending on the spatial arrangement of materials [16,17]. Three-dimensional (3D) DNA nanostructures have been applied to encapsulate enzymes and nucleic acids [18,19,20,21,22,23]. However, the molecules assembled inside the 3D scaffold would not be well isolated from the external environment because ions and small molecules would pass through the gap of DNA nanostructure. To isolate the materials assembled on a DNA scaffold from the external environment efficiently, further encapsulation of the molecules assembled on DNA scaffold is required.

Yang et al. [24] reported using a ring-shaped DNA nanostructure [25] to prepare a defined size of liposome inside the ring. While the method is attractive, the ring-shaped DNA does not provide a platform to locate molecules of interest inside the liposome. In this study, a ring-shaped DNA nanostructure was appended with an inner bridge of double-stranded DNA as a platform that enables assembly of molecules of interest inside the ring. The bridged ring-shaped DNA nanostructure successfully served as an outer skeleton to prepare a liposome while encapsulating the inner DNA bridge. The bridge was modified with a pH-sensitive fluorophore [26] to monitor the local pH with or without the liposome formation. Without the liposome formation, the emission of the pH-sensitive fluorophore on the bridge platform responded to the pH change of the buffer, but it did not respond to the pH change upon formation of the liposome guided by the ring skeleton to indicate a successful formation of a compartment. The bridged DNA duplex would provide a convenient platform to specifically locate molecules of interest in the encapsulated environment to develop functional artificial compartments.

## 2. Results and Discussions

### 2.1. Design of a Ring-Shaped DNA Origami Skeleton with a Bridged DNA Platform

**Figure 1:**
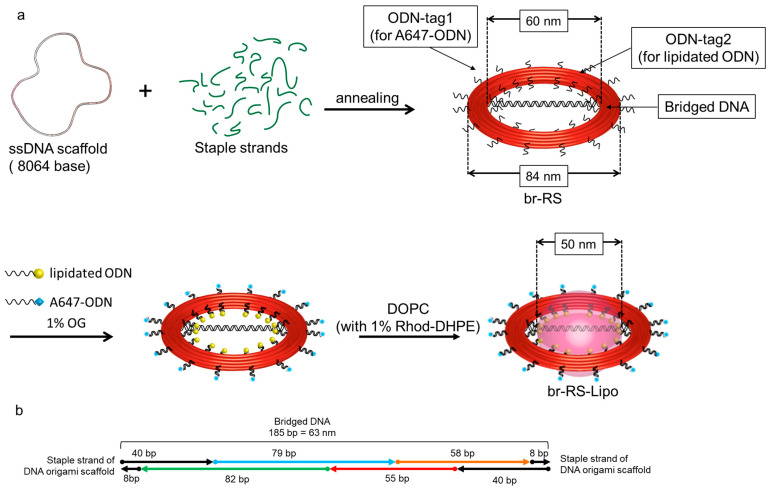
Design of a bridged ring-shaped DNA origami skeleton that guides formation of a nanoliposome. (**a**) Schematic representation of the ring-shaped DNA origami skeleton with bridged DNA (br-RS), br-RS modified with lipidated ODN, and br-RS guided liposome (br-RS-Lipo). See also Figure S1. (**b**) Representative design of the bridged DNA. See also Figure S1.

**Figure 2:**
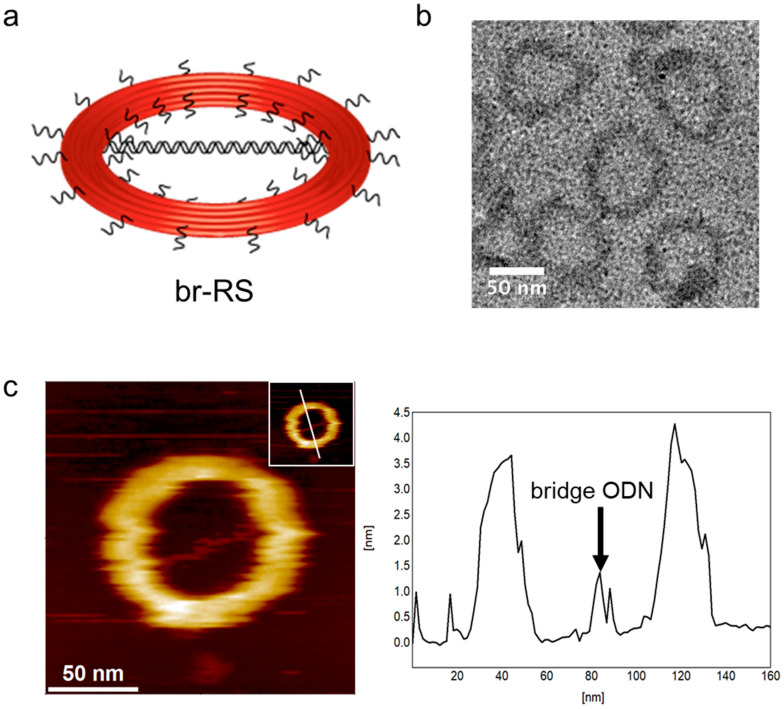
Structural characterization of br-RS. (**a**) An illustration of br-RS. (**b**) A TEM image of br-RS-1. (**c**) An AFM image of br-RS-1 (**left**). Inset: a line indicates the cross-section of image for the height analysis (**right**). An arrow indicates the bridge ODN of br-RS-1 (**right**).

A ring-shaped DNA origami skeleton (RS) with a 60-nm inner diameter and an 84-nm outer diameter was designed as previously described [24]. The DNA ring was bridged by a double-stranded DNA platform (185 bp, 63 nm) consisting of four oligo DNAs (ODNs) modified with fluorophore Alexa488 (A488) or 6-carboxyfluorescein (CF) (Figure 1, Supplementary Materials Figure S1 and Table 1) to prepare a bridged DNA ring skeleton (br-RS-1). The outer and inner handles for attachment of the fluorophore (Alexa647)-modified ODN (A647-ODN) and lipidated ODN were also designed in orthogonal nucleotide sequences on RS (Figure S1). br-RS-1 was folded by mixing a long single-stranded DNA (8064 nucleotide) and staple strands (Table S1) in a molar ratio of 1:6 followed by a thermal annealing step for 35 h. The folded br-RS-1 was purified by size exclusion chromatography (Sephacryl S-400) to remove the excess staple stands and characterized by means of atomic force microscopy (AFM) and transmission electron microscopy (TEM) (Figure 2). The sizes of br-RS-1 were 59.6 ± 3.3 nm (inner diameter) and 83.2 ± 3.9 nm (outer diameter) in the TEM images, which were consistent with the designed sizes (Figure 1a) and previous reports [24]. The bridged ODN inside the RS was confirmed by AFM images (Figure 2c). The yield of well-formed br-RS-1 containing the bridged DNA platform was determined to be 31% from the AFM images. 

### 2.2. Construction of a Nanoliposome Guided by the DNA Origami Ring Skeleton to Encapsulate the Bridged DNA Platform

A647-ODN and lipidated ODN (Figure S1), which have complementary nucleotide sequences with the outer and inner handles, respectively, were assembled on br-RS. The lipidated ODN was prepared as described previously (Figure S2) [24]. A thiol-modified ODN complementary to the inner handle of br-RS was conjugated with maleimide modified DOPE via a thiol-maleimide crosslinking reaction. br-RS was mixed with an excess amount of A647-ODN and lipidated ODN in a solution containing 0.67% *n*-octyl-b-D-glucopyranoside (OG). br-RS-1 thus labeled by fluorophore (A647) and lipid (DOPE) was mixed with lipid (DOPC) containing 1% NBD-modified lipid (NBD-DPPE) or 1% rhodamine (Rho)-modified lipid (Rho-DHPE) (Table 1). The solution was dialyzed against a buffer (pH 7.5) containing 25 mM HEPES, 10 mM MgCl_2_, and 0.4 M KCl for 16 h to remove detergent and form the liposome. The solution was loaded onto an iodixanol gradient and spun in an ultracentrifuge to separate a nanoliposome guided by DNA origami ring (br-RS-1-Lipo). The fluorophore attached to each module of br-RS or br-RS-Lipo, i.e., RS, bridged DNA, and lipid, are given in Table 1 and Figure S1.

Firstly, agarose gel electrophoretic analysis was conducted to characterize the separated fractions of crude br-RS-1-Lipo, which contained br-RS, A647-ODN, and lipidated ODN, and DOPC with NBD-DPPE (1%) (Figure S3a and Table 1). A sample contained all the components for preparation of br-RS-1-Lipo, but using br-RS-1 lacking the lipidated ODN, was also prepared for comparison (Figure S3b). Fractions collected after the ultracentrifugation were loaded on an agarose gel containing 0.05% SDS to decompose the liposome (Figure S3a). The initial fractions (F1-3) revealed only a band with the fluorescence emission of NBD, indicating that these fractions contained only the lipid. In the following fractions (F4-7), both a band corresponding to br-RS-1 with the fluorescence emissions from A647 (RS) and the band of lipid-emitting NBD (lipid) were observed, indicating the presence of br-RS-1-Lipo. The denser fractions (F8-13) revealed the bands corresponding to small amount of br-RS-1 and lipid. In contrast, the band corresponding to br-RS-1 appeared mainly in the denser fractions (F10-13) and the band corresponding to lipids appeared only in the initial fractions (F1-5) in the sample prepared in the absence of lipidated ODN on RS (Figure S3b). Without the addition of lipidated ODN, br-RS and lipid did not coexist in the same fraction. These results indicated the specific formation of liposome guided by br-RS. The bridged DNA platform did not interfere with the liposome formation. 

**Figure 3:**
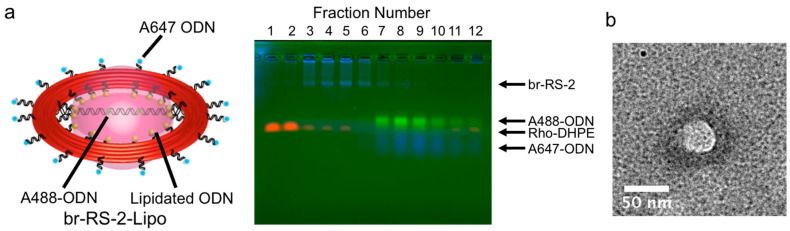
Formation of nanoliposome encapsulating the bridged DNA platform guided by the DNA origami ring skeleton. (**a**) Agarose gel image of the density gradient fractions of the crude mixture of br-RS-2-Lipo. Fractions are numbered sequentially from F1 to F13 from the top to the bottom of gradient. The fluorescence emissions of A647 on RS, A488 on bridged DNA, and Rho on lipid were represented as blue, green, and red, respectively. RS (A647) and lipid (Rho) were separated in the presence of 0.05% SDS. The same agarose gel image separately visualized by each fluorophore was shown in Figure S4. (**b**) A TEM image of the fractions containing br-RS-2-Lipo (F4 and F6 in Figure 3a). The sizes of br-RS and liposome were 73.6 ± 4.4 nm in outer diameter and 41.3 ± 5.6 nm, respectively. The scale bar: 50 nm.

In order to confirm the presence of the bridged DNA platform, br-RS-2-Lipo was prepared by br-RS-2, which contained A488-modified bridge ODN, A647-ODN and lipidated ODN, and DOPC with Rho-DHPE. The crude mixture of br-RS-2-Lipo was loaded onto an iodixanol gradient, spun in an ultracentrifuge and the separated fractions were loaded on an agarose gel containing 0.05% SDS (Figure 3a). As observed in the case of br-RS-1-Lipo, the initial fractions (F1-2) contained only lipids. The following fractions (F3-5) contained the band corresponding to br-RS-2 with the fluorescence emissions of A647 (RS) and A488 (bridged DNA) and lipid showing the emission of Rho (Figure S4). These results confirmed isolation of br-RS-2-Lipo consisting of the DNA-bridged DNA origami ring and lipid. 

The fractions containing br-RS-2-Lipo were analyzed by transmission electron microscopy (TEM) to identify the DNA ring and liposome. As shown in Figure 3b, a typical TEM image of br-RS-2-Lipo showed a bright white circle in the center of a black ring by staining with Ti blue. By comparing the images of br-RS-1 alone (Figure 2b) with a previous report [24], the black ring (outer diameter: 73.6 ± 4.4 nm) and white circle (diameter: 41.3 ± 5.6 nm) were identified as br-RS and nanoliposome, respectively. 

### 2.3. Controlled Assembly of Fluorophores on the Bridged DNA Platform Encapsulated in the Nanoliposome 

**Figure 4:**
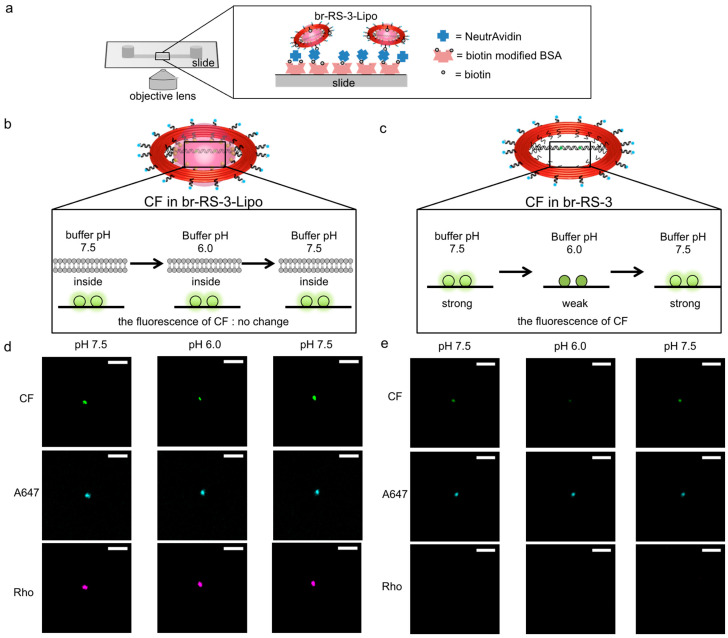
Fluorescent microscopic images of fluorophore modified br-RS-3-Lipo and br-RS-3. (**a**) An illustration showing immobilization and measurement system of br-RS-3-Lipo or br-RS-3. Biotin modified on br-RS-3-Lipo or br-RS-3 bound to NeutrAvidin that attached to a biotinylated BSA pretreated slide (µ-Slide I^0.2^ Luer ibidi Treat). (**b**,**c**) Illustrations of the fluorescent intensity change of (**b**) br-RS-3-Lipo or (**c**) br-RS-3 alone (without lipidated DNA) by changing buffer pH. (**d**,**e**) Fluorescent images of CF (bridged DNA), A647(DNA ring) and Rho(liposome) for (**d**) br-RS-3-Lipo or (**e**) br-RS-3 alone. In the case of (**e**) br-RS-3 alone, no spot was observed in the Rho channel. CF channel was monitored at 535 nm when excited at 482 nm. A647 channel was monitored at 710 nm when excited at 654 nm. Rho channel was monitored at 610 nm when excited at 568 nm. The scale bar shows 100 µm.

To test the controlled assembly of molecules of interest on the bridged DNA platform and to verify their encapsulation in the nanoliposome compartment, a pH-sensitive fluorophore was assembled on the bridged DNA platform and the nanoliposome was analyzed by means of fluorescent microscopy. br-RS-3-Lipo was prepared with RS labeled by A647, CF-assembled bridged ODN, and lipid labeled by Rho (Table 1). To immobilize br-RS-3 and br-RS-3-Lipo on a microscope slide, RS was incorporated into a biotin-modified DNA (see Figure 4a and Figure S1). The prepared br-RS-3-Lipo was purified as described in the previous section (see Section 2.1 and Figure S5). The br-RS-3 and br-RS-3-Lipo were immobilized on a microscope slide through the biotin-avidin interaction (see Materials and Methods and Figure 4a). Under microscopic observation (Figure S6), the fluorescence emissions of CF, Rho, and A647 coexisted at the same position of the fluorescent image of br-RS-3-Lipo (75%) as defined by pattern (iii). Other spots revealed coexistence of the fluorescence emission of Rho and A647 (17%) as the categorized pattern (ii), which indicated the presence of RS-Lipo. When br-RS-3 was utilized as the skeleton, most of the spots revealed the coexistence of fluorescence emissions of CF and A647 (81%) as pattern (v) or only the emission of A647 alone (19%) as pattern (iv). 

Encapsulation of the bridged DNA inside the nanoliposome was verified by using the sensitivity of CF emission to pH changes [26]. The pH titration of br-RS-3 was conducted in a test tube (Figure S7) and the plot gave an estimation of p*K*_a_ of CF on br-RS (6.9 ± 0.2). The estimated p*K*_a_ value was consistent with that of CF on a DNA nanostructure as reported previously [27]. The result indicated that the fluorescence emission of CF on the bridged platform retained its sensitivity to pH changes and was effectively decreased at pH 6.0. Fluorescent microscopic analyses were carried out by changing the pH of outer buffer of the immobilized liposome (Figure 4b–e). At first, the immobilized br-RS-3 without lipidated DNA, of which CFs on the bridge platform were exposed to the bulk solution, was analyzed at pH 7.5. In that case, CF showed strong fluorescence as shown in Figure 4e (left). When the buffer was changed to pH 6.0, the fluorescence of CF drastically reduced to 62% of initial fluorescence intensity, whereas the A647 showed little or no change (93% of initial fluorescence) (Figure 4e, middle). By changing the buffer again to pH 7.5, the reduced fluorescence of CF at pH 6.0 recovered to 92% of the initial fluorescence intensity at pH 7.5 (Figure 4e, right). The result indicated CF on the br-RS-3 responded well to the pH change of the bulk buffer solution (Figure 4c). On the other hand, the intensity fluorescence emission of CF of br-RS-3-Lipo did not show a significant difference during the sequential change of outer buffer from pH 7.5 to pH 6.0 (91% of initial fluorescence intensity) (Figure 4d, middle), then back to pH 7.5 (almost 100% of initial fluorescence) (Figure 4d, right). The result strongly indicated that the bridged DNA platform on RS was encapsulated inside the nanoliposome and the local environment at the platform was well-sealed from the outer pH change (Figure 4b). Not surprisingly, some of the br-RS-3-Lipo spots showing all the fluorescence of CF, Rho, and A647 revealed the fluorescence intensity change of CF during the pH change of outer buffer (see Figure S8 and note). That might be the result of insufficient encapsulation of the CF-contained bridged DNA platform because of the partial degradation of nanoliposome. One of the possible factors causing the degradation of liposome compartments is the instability of ring-shaped skeleton RS, which tends to be linearized at certain conditions, such as the AFM measurements. We have reported that ligation of staple strands in DNA nanostructures effectively increases their stability [28]. The ring-shaped skeleton RS, together with the bridged DNA platform, would be stabilized by applying our ligation reaction, which in turn increases the overall stability of liposome compartments. 

## 3. Materials and Methods

### 3.1. Materials

Purified oligonucleotides, as the staple strands for DNA origami and all other oligonucleotides, were purchased from Sigma-Aldrich (St. Louis, MO, USA) or Thermo Fisher Scientific Inc. (Waltham, MA, USA) Ultrafree-MC-DV and Amicon Ultra-0.5 mL Centrifugal Filters were purchased from Merck Millipore (Darmstadt, Germany). Lissamine™ rhodamine B DHPE, triethylammonium salt (Rho-DHPE), biotinylated Bovine Serum Albumin, and NeutrAvidin were purchased from Thermo Fisher Scientific Inc. (Waltham, MA, USA). 1,2-Dioleoyl-sn-glycero-3-phosphocholine (DOPC) was purchased from Sigma-Aldrich (St. Louis, MO, USA). N-(3-Maleimide-1-oxopropyl)-L-α-phosphatidylethanolamine, Dioleoyl (DOPE-MAL) was purchased from NOF CORPORATION (Tokyo, Japan). N-Octyl-b-D-glucoside (OG), HEPES and MES were purchased from DOJINDO (Kumamoto, Japan). Opti Prep (60% iodixanol solution) was purchased from Axis-Shield Diagnostics Ltd. (Dundee, England). TI blue was purchased from Nissin EM Co., Ltd. (Tokyo, Japan). A 15-nm carbon grid U1017 from EM Japan (Tokyo, Japan) was used for TEM grid. µ-Slide I^0.2^ Luer ibidi Treat was purchased from ibidi (Gräfelfing, Germany). Low-binding microtubes (BT-150L, 1.5 mL, nonpyrogenic and RNase-/DNase-free) were purchased from Ina OPTIKA CO., LTD (Osaka, Japan). The 384 Well Low Volume Plate Black Round-Bottom NBS was purchased from Corning (Corning, NY, USA). Other chemical compounds were purchased from FUJIFILM Wako Pure Chemical Corporation (Osaka, Japan) or Nacalai Tesque (Kyoto, Japan). 

### 3.2. Preparation of the DNA Scaffold

A solution (50 µL) containing 8064 base single-stranded DNA (10 nM) and a mixture of staple DNA strands (50 nM) (Table S1) in a buffer (pH 8.0) containing 40 mM Tris–HCl, 20 mM acetic acid, and 12.5 mM MgCl_2_ was heated at 95 °C for 1 min, incubated at 80 °C for 5 min, cooled down to 65 °C for 75 min, incubated at 65 °C for 20 min, cooled down to 25 °C for 2000 min and then cooled down to 15 °C by using a thermal cycler (C1000 Thermal Cycler, BioRad, Hercules, CA, USA). The sample was purified by gel filtration (500 mL in volume of Sephacryl S-400) in an Ultrafree-MC-DV column to remove excess staple strands. The concentration of the DNA scaffold was quantified by absorbance at 260 nm (Nanodrop, Thermo Fisher Scientific Inc., Waltham, MA, USA) using the determined extinction coefficient of the DNA scaffold (1.2 × 10^8^ M^−1^ cm^−1^).

### 3.3. Preparation of Liposome on DNA Nanostructure

A solution containing 10 nM of DNA nanostructure, 240 nM of Alexa Fluor 647 modified DNA, 480 nM of lipid modified DNA and 0.67% OG in 25 mM HEPES, 10 mM MgCl2 was incubated at 37 °C for 1 h. To form DNA-ring templated liposomes, 15 µL of 25 mM HEPES, 10 mM MgCl_2_ (pH 7.5), 2 µL of 6.7% OG, 6 µL 4 M KCl and 5 µL of 15 mM DOPC (1% Rhod-PE) were added to 40 µL of 10 nM lipid- and Alexa Fluor 647-labeled DNA rings to reach 60 µL total volume and the solution was shaken for 30 min at room temperature. The solution was diluted with 60 µL of 25 mM HEPES, 10 mM MgCl_2_ and 0.4 M KCl buffer containing 0.67% OG and dialyzed against 2 L of buffer overnight. The amount of 100 µL of recovered solution was mixed with 200 µL of 30% iodixanol in 1× hydration buffer and placed at the bottom of a centrifuge tube (11 × 34 mm, Beckman Coulter Inc., Brea, CA, USA). Six additional layers of 0 to 15% iodixanol solution in buffer were added to the centrifuge tube. The tube was spun in an MLA-130 rotor (Beckman Coulter Inc. Brea, CA, USA) at 237,000× *g* and 4 °C for 5 h, then after the fractions (52 μL per fraction) were collected.

### 3.4. Synthesis of Lipidated DNA

A volume of 400 µM of Thiol-modified DNA oligonucleotides were treated with 10 mM TCEP (tris(2-carboxyethyl)phosphine) in 40 mM phosphate buffer (pH 8.0) for 30 min at room temperature and reacted with 8 mM of DOPE-MAL containing 2% OG overnight. The reaction mixture was purified by reverse-phase HPLC on a Cosmosil 5C18-MS II column (4.6 × 150 mm, eluted with 0.1 M TEAA (triethylammonium acetate) buffer, pH 7.0, with a liner gradient over 10 min from 52 to 75% acetonitrile at a flow rate of 1.0 mL·min^−1^) and characterized by MALDI-TOF mass spectrometry (AXIMA-LNR, Shimadzu, Kyoto, Japan) (HPA matrix). Lipidated ODN: *m*/*z* calcd 7637, observed 7640.

### 3.5. Agarose Gel Electrophoresis

The samples were run on a 1.5% agarose gel in 0.5 × TBE containing 10 mM MgCl_2_ and 0.05% SDS at 50 V for 6 h in the cold storage chamber. The gel was visualized by using ChemiDoc™ MP (Bio-Rad, Hercules, CA, USA) under Alexa 488 or CF channel (λ_ex_ = 475 nm, λ_em_ = 532 nm), Rho (λ_ex_ = 532 nm, λ_em_ = 595 nm), or Alexa 647 channel (λ_ex_ = 637 nm, λ_em_ = 700 nm).

### 3.6. AFM Imaging

The purified DNA nanostructure was deposited on a mica (1.5 mm in diameter) surface, adsorbed for 5 min at ambient temperature, and then washed three times with a buffer (pH 8.0) containing 40 mM Tris–HCl, 20 mM acetic acid, and 12.5 mM MgCl_2_. The sample was scanned in solution with tapping mode using a fast-scanning AFM system (Nano Live Vision, RIBM Co. Ltd., Tsukuba, Japan) with a silicon nitride cantilever (BL-AC10DS-A2, Olympus, Tokyo, Japan). At least three independent preparations of each sample were analyzed by AFM, and several images were acquired from different areas of the mica surface. 

### 3.7. TEM Imaging

The sample, after removing iodixanol by Amicon Ultra-0.5 mL Centrifugal Filters (30-kD NMWL) (2 µL), was placed onto a glow-discharged TEM grid and incubated for 2 min. Next, the extra sample was removed by filter paper. Next, 15 µL of MilliQ water was used for washing the surface of the TEM grid, followed by incubation with TI blue (2 µL) for 2 min. The surface was washed by MilliQ water. Samples were analyzed by using a TEM microscope (JEOL JEM-2200FS + CETCOR, Tokyo, Japan).

### 3.8. Fluorescent Microscopy

The DNA nanostructure containing biotin modified staple strands was loaded to a µ-Slide I^0.2^ Luer ibidi-Treat pretreated by biotinylated bovine serum albumin and neutravidin, adsorbed for 15 min at room temperature, and then washed with a buffer (pH 7.5) containing 25 mM HEPES, 10 mM MgCl_2_, and 0.4 M KCl. The fluorescence of the samples was observed using an IX-81 fluorescence microscope (Olympus, Tokyo, Japan) equipped with a 20× objective lens and Xe lamp. Fluorescence images were acquired with electron multiplier CCD camera (Hamamatsu Photonics K.K., Shizuoka, Japan) at ambient temperature. After the fluorescence of fluorescein, rhodamine, and Alexa Fluor 647 were sequentially monitored, the sample was washed with a buffer (pH 6.0) containing 25 mM HEPES, 10 mM MgCl_2_, and 0.4 M KCl. The fluorescence images of them were taken at the same position. After that, the sample was washed again with a buffer (pH 7.5) containing 25 mM HEPES, 10 mM MgCl_2_, and 0.4 M KCl, and the fluorescence images were taken. 

### 3.9. Fluorescence Measurement 

The fluorescence intensity of CF and Rho on DNA nanostructures was measured at 25 °C by an Infinite 200 PRO microplate reader (TECAN, Männedorf, Switzerland) with excitation at 480 nm and 620 nm in the 40 mM acetate (pH 5.5), MES (pH 6.0 to 6.5) or HEPES (pH 7.0 to 8.0) buffer containing 12.5 mM MgCl_2_, respectively. Fluorescence measurements were performed in 384-well plates.

## 4. Conclusions

In this study, a ring-shaped DNA origami skeleton was utilized for liposome formation and a double-stranded DNA was bridged on the ring to provide a platform to introduce molecules of interest inside the liposome. Formation of the ring skeleton and bridged platform was confirmed by means of agarose gel electrophoretic analyses, AFM and TEM measurements. Compartmentalization upon the nanoliposome formation was verified by assembling a pH-sensitive fluorophore on the bridged DNA platform. The pH-sensitive fluorophore 6-carboxyfluorescein attached to the bridged DNA showed a null response to the pH change of outer buffer upon formation of the ring skeleton-guided liposome. The nanoliposome encapsulating a platform to assemble molecules of interest would be applied for the pH endoscope [27], in which two types of fluorophores are assembled on a DNA nanostructure to realize ratiometric fluorescent pH monitoring during its internalization process to the cell by fluorescence microscope measurements. Assembling appropriate number of the same type of fluorophore within nanoliposome would increase the emission intensity at resolution limit. Likewise, combination of multiple types of probes within the nanoliposome would enable simultaneous monitoring of environmental factors when loaded into the cell. 

It should be noted that liposomes are commonly labelled with fluorophores by incorporating fluorophore-labelled lipids into the liposomal membrane or by involving fluorophores within the inner aqueous phase of the liposome [29]. However, these methods suffer from the difficulty of strictly controlling the number, stoichiometry, and location of fluorophores loaded to liposomes. DNA nanostructure-based scaffolds are quite useful for controlling these factors owing to their accurate addressability [30]. Though the stability of liposome and/or bridged-RS and the yield of encapsulated fluorophores inside RS need to be improved as described above, these issues can be addressed by applying our nick-ligation technique of DNA origami [28]. Upon ligation, the bridged DNA should be a useful platform to assemble not only the pH-sensitive fluorophore as shown in this study, but also other functional molecules introduced on the DNA nanostructures previously [31]. For example, enzymes of interest can be located on the bridged DNA to encapsulate a defined number of enzymes within a nanoliposome by using the modular adaptors developed by our group [32,33,34,35,36,37]. The bridged ring skeleton with spatially assembled enzymes would be converted to a compartment upon the formation of liposome. Studies on the cascade reactions with defined number of enzymes inside the nanoliposome would provide chemical information necessary to understand the biological cascade reaction in the compartment such as the cellular organelles and to construct artificial metabolic systems. Work in this direction is ongoing in our laboratory.

## Figures and Tables

**Table 1:** Fluorophores assembled at the designated module of br-RS and br-RS-Lipo.

	Modules of br-RS or br-RS-Lipo		
	RS	Bridged DNA	Lipid
br-RS-1	A647	none	none
br-RS-1-Lipo			NBD
br-RS-2	A647	A488	none
br-RS-2-Lipo			Rhodamine (Rho)
br-RS-3	A647	Fluorescein (CF)	none
br-RS-3-Lipo			Rho

## Data Availability

Not applicable.

## Supplementary Materials

The following supporting information can be downloaded at: https://www.mdpi.com/article/10.3390/molecules28020911/s1, Figure S1: Detail of the design of a bridged ring shaped DNA origami skeleton and the modifier; Figure S2: Synthetic scheme of lipidated DNA; Figure S3: Characterization of the nanoliposome encapsulating br-RS; Figures S4 and S5: Agarose gel images; Figures S6 and S8: Fluorescent microscopic images; Figure S7: pH titration of br-RS-3; Table S1: Nucleotide sequences for the staple strands.

## Sample Availability

Samples of the compounds are available from the authors upon request.
