# Supplementary material for: Controlled Assembly of Fluorophores inside a Nanoliposome
Source: Molecules. 2023 Jan 16;28(2):911. doi: 10.3390/molecules28020911 (PMC9864194; doi:10.3390/molecules28020911)
Supplement: Supplementary file 1 [file molecules-28-00911-s001.zip › molecules-2138528-supplementary.pdf]

-SUPPORTING INFORMATION-

Controlled assembly of fluorophores inside the nanoliposome

Hiroaki Konishi, Eiji Nakata, Futa Komatsubara, and Takashi Morii *

Institute of Advanced Energy, Kyoto University, Uji, Kyoto 611-0011, Japan.

*To whom correspondence should be addressed:

Prof. Takashi Morii

Tel.: +81 774-38-3515

Fax: +81 774-38-3516

E-mail: t-morii@iae.kyoto-u.ac.jp

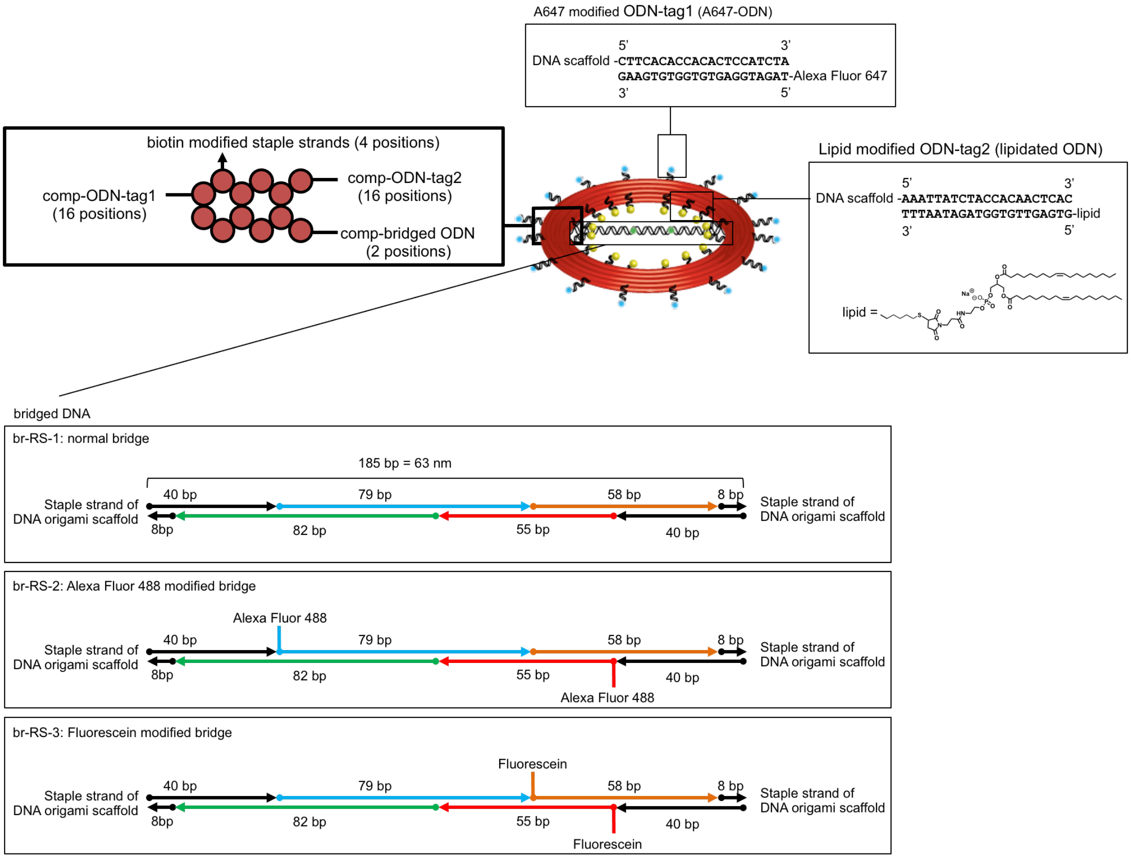

Figure S1. Detail of the design of a bridged ring shaped DNA origami skeleton and the modifier (A647-ODN, lipidated ODN, bridged DNA and biotin modified staple strands).

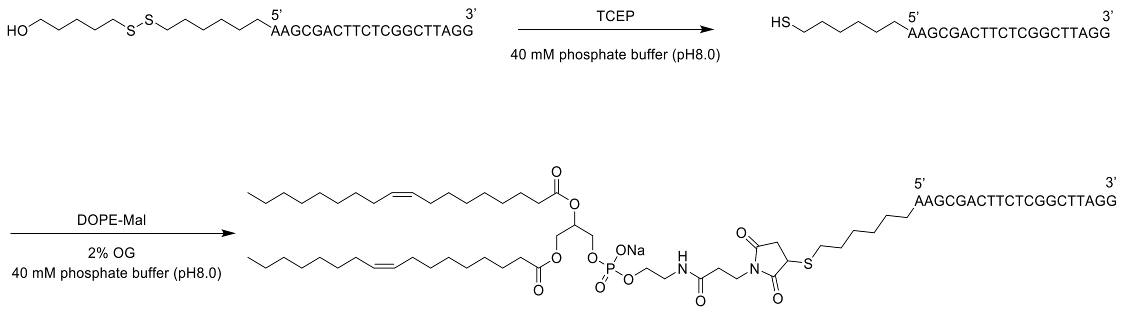

Figure S2. Synthetic scheme of lipidated DNA.

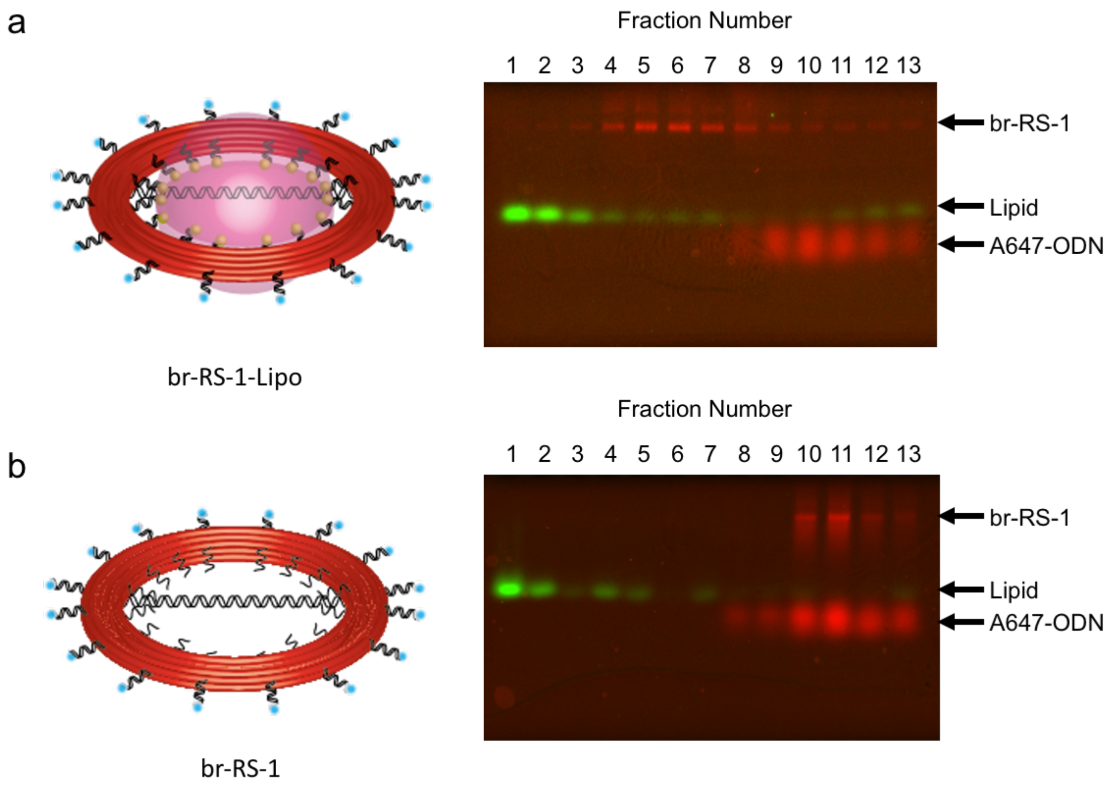

Figure S3. Characterization of the nanoliposome encapsulating the bridged DNA platform guided by the DNA origami ring skeleton. (a and b) Agarose gel images of the density gradient fractions of br-RS (a) with lipidated DNA (br-RS-1-Lipo) or (b) without lipidated DNA (br-RS-1). Fractions are numbered sequentially from F1 to F13 from the top to the bottom of the gradient. A647 labeled br-RS (red) and NBD labeled lipid (green) were separated in the presence of 0.05% of SDS.

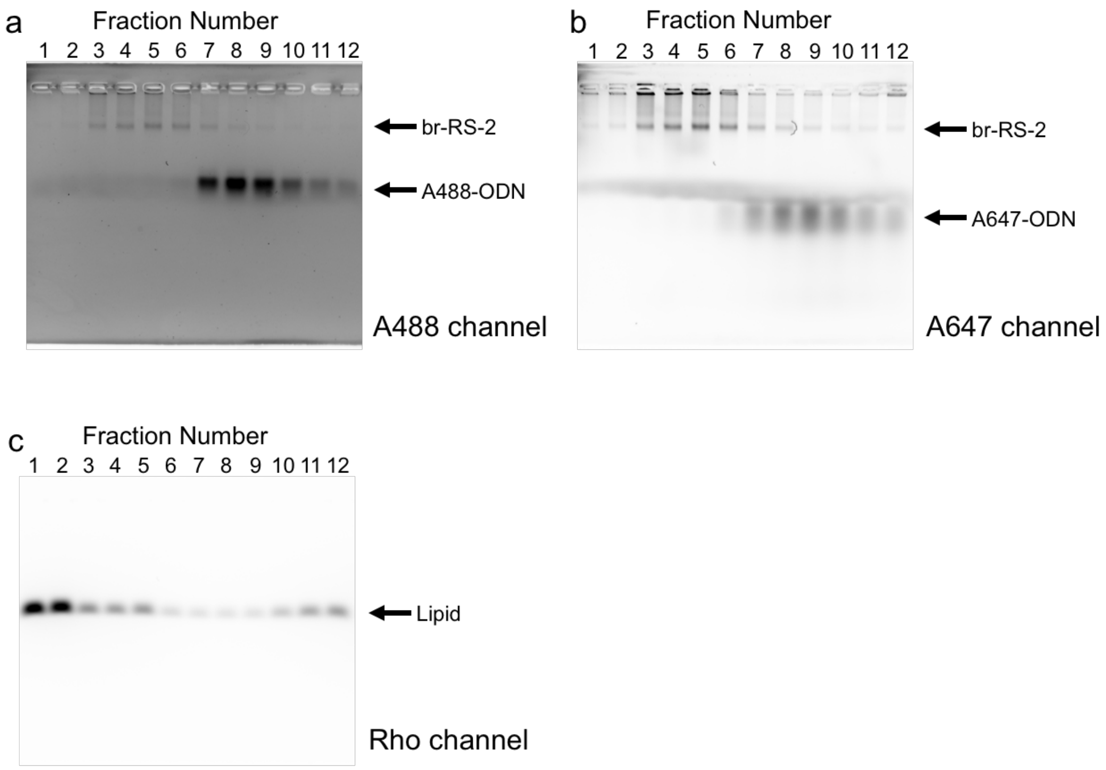

Figure S4. Agarose gel images of the density gradient fractions of br-RS-2-Lipo visualized by (a) A488, (b) A647 and (c) Rho fluorescence. The merged image was shown in Figure 3.

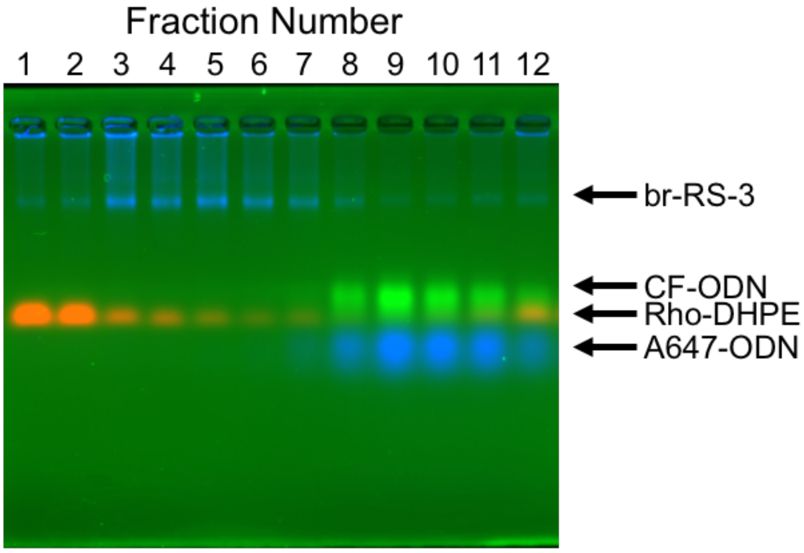

Figure S5. Agarose gel image of the density gradient fractions of br-RS-3-Lipo visualized by CF (green), A647 (blue) and Rho (red) fluorescence.

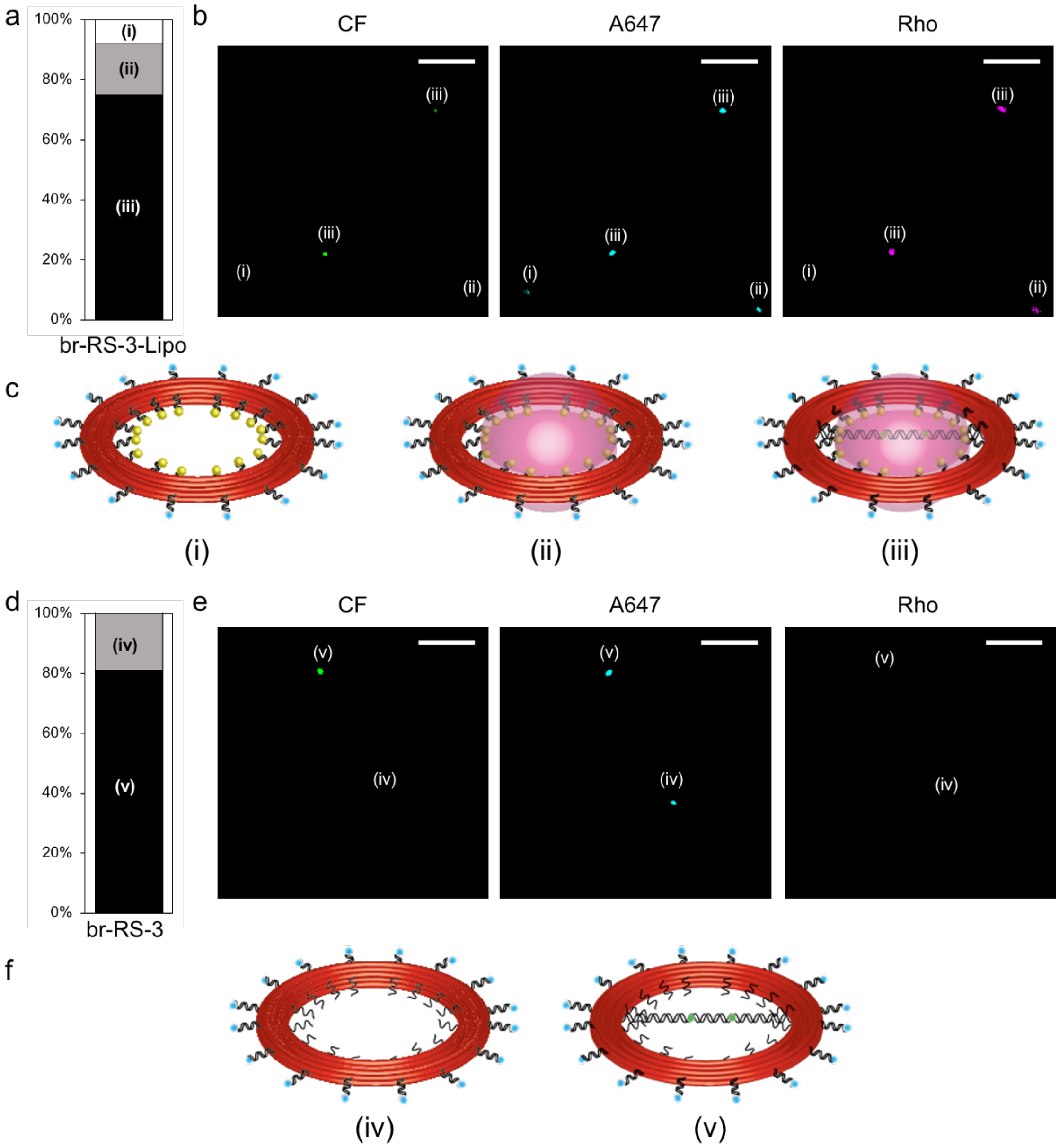

Figure S6. (a) The percentages of the combination of the fluorescence observed at the same position in the fluorescent images of br-BS-3-Lipo. (i), (ii), and (iii) indicated the spot having the fluorescence of A647 alone, A647 and Rho without CF, and all of them, respectively. (b) A typical fluorescent image of br-RS-3-Lipo in the CF, A647 and Rho channels at pH 7.5. (c) The possible cartoons observed as (i), (ii), and (iii). (d) The percentages of the combination of the fluorescence observed at the same position in the fluorescent images of br-BS-3. (iv) and (v) indicated the spot having the fluorescence of A647 alone and both of A647 and CF, respectively. (d) A typical fluorescent image of br-RS-3 in the CF, A647 and Rho channels at pH 7.5. (e) The possible cartoons observed as (iv), and (v). The scale bar indicated 200 μm.

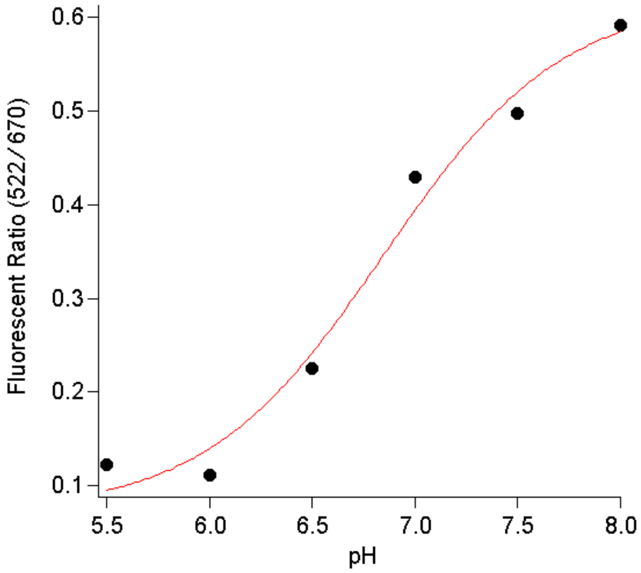

Figure S7. pH titration of br-RS-3. A plot for the ratio of emission intensity at 522 nm excited at 480 nm, corresponding to CF, to that at 670 nm excited at 670 nm, corresponding to A647 at various pH in the range from 5.5 to 8. The emission intensity ratios (522/670) of each pH were plotted. The pK_a value was estimated to be 6.9 ± 0.2 at ambient temperature.

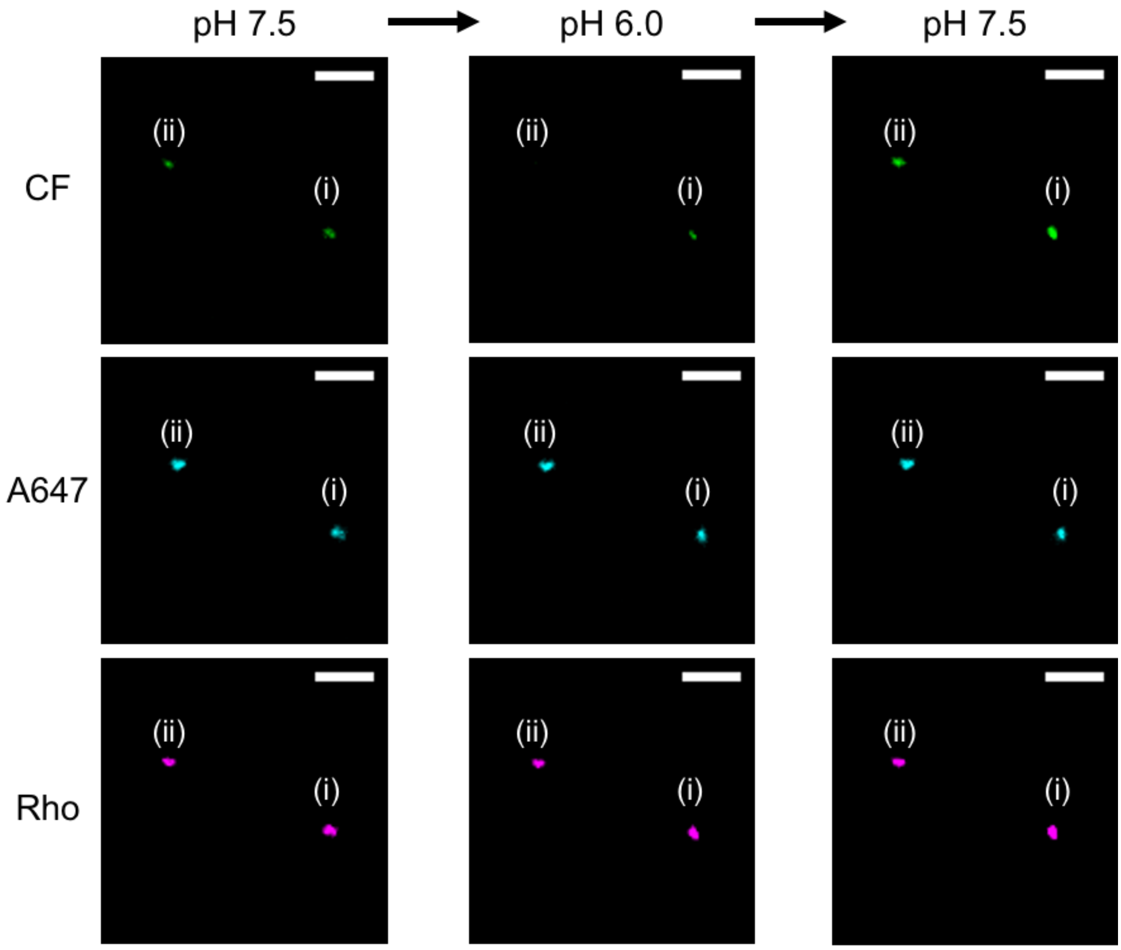

Figure S8. A typical fluorescent image of br-RS-3-Lipo in the CF (br), A647 (RS) and Rho (liposome) channels. The scale bar indicated 100 μm.

Note: In this image, two types of br-RS-3-Lipo (i and ii) were included. Though both of them have all the fluorescence of CF, Rho, A647 at initial pH7.5 (left), they showed different response toward outer pH change. Under the observation, the fluorescence of A657, and Rho were almost constant. In the CF channel, one (i) showed no response toward outer pH change. That is, the CF on RS was successfully encapsulated inside a nanoliposome. On the other hand, the other (ii) showed significant fluorescence decrement in the CF channel at pH6.0 (middle), and the fluorescence was recovered after back to pH7.5 (right). The result indicated the failure of the encapsulation of CF-contained bridged DNA.

Table S1. Nucleotide sequences for the staple strands

DNA sequences of A647-ODN and thiol modified ODN for preparation of lipidated ODN	
Oligo DNA	Sequence (from 5' to 3')
Alexa Fluor 647 modified DNA	Alexa Fluor 647-TAGATGGAGTGTGGTGTGAAG
thiol modified DNA	thiol - GTGAGTTGTGGTAGATAATTT

staple strand for bridge DNA	
bridge-01	CCACCCTGCGGGTGCGCCAACGCGCAACGTTACTCAGTATGGATCG AGCTCGCCTCTAGATGCGGGTGCGCCAATCTCA
A488-bridge-01	A488- CCACCCTGCGGGTGCGCCAACGCGCAACGTTACTCAGTATGGATCG AGCTCGCCTCTAGATGCGGGTGCGCCAATCTCA
bridge-02	CAAGTTACTCAGTATCGAACCGGCACTTAAGTTCCGCCACCCACGCG CAACGAGGAGT
CF-bridge-02	CF- CAAGTTACTCAGTATCGAACCGGCACTTAAGTTCCGCCACCCACGCG CAACGAGGAGT
bridge-03	GTGCCGGTTCGATACTCAGTAACTTGTGAGATTGGCGCACCCGCATC TAGAGGCG
A488-bridge-03	A488- GTGCCGGTTCGATACTCAGTAACTTGTGAGATTGGCGCACCCGCATC TAGAGGCG
CF-bridge-03	CF- GTGCCGGTTCGATACTCAGTAACTTGTGAGATTGGCGCACCCGCATC TAGAGGCG
bridge-04	AGCTCGATCCATACTGAGTAACGTTGCGCG TTGGCGCACCCGCAGG GTGGCGGAACTTAAGTGCCGGTAGTCCTTGTGAGAT

other staple strands	
1	GCTATTTACACAGACAATAAACATGCGCTCGTGGACTC
2	GCCAACGCCAGGGTGGTTGTAAGAATACGTGGCCCCTCTGA
3	ACCACCATCGCCATACTAATACCATTGCAACAGGAAAAA
4	TTTCACCCCTGGCCCTGAGACAGAGATAGAACCCTGCAAGGGACA

5	CCTGAAAGAGTCTGAAATGGA
6	GTGCCACCGGTCAGTTGAAAGGAGCACTAAATTT
7	ATTTGCTGAACCTCAACAAATCAACACATTGGCAGATT
8	TGTTTGAAAGAGTTGCAGCAAGCGGTCCACGCTGGTAGGATTGCCC
9	CACCAGGTGGTTCCGAGCCCGAGCTTTCT
10	TTATTTAGTATTAACAGAACGA
11	TGACGCTCAATCTAGGGTTGAATTAAAGTTAATCG
12	GAAATACCTACCATAAAAAAGTCTT
13	CGCCAGGATTAGTCTTTAGGAATTGATCAGTTGGATATCAAA
14	GGAAACCGTCACTGCCCGCCGAGCTCTATCAGGGCGAT
15	CTTTGCTCAGCAAATCGTTAATTGCGTTGCGCGTAGGAGCT
16	ATTGGGCGCGCGGGACAAGAGCAAAGGGCGAAA
17	AACTCACCTTTCCTGTGTGAAATTGTTTACGGGCAACTTGCGT
18	GCATCAGCCCCTGCATCAGAGGGTGCCTAATGAGTCTCATAAAGT
19	GGATTGCCCCAGCAGGCAAAATCCCTATCCGCTCACAAT
20	CCAGAATTGCGATCCAGCGCAGTGTCACTGCGCGCCCTCAGCGGTG
21	TCCACAGGCGGGCCGCGCGTGCATTAACG
22	GGTCATAGCTGTGTTCTTCGCCGGGTACTTGGTTT
23	CAACGTTCCACTGTGTTGTAAAGAATAAATCGGCGAAAATCC
24	AACCGTCGAATTCGTAAGAGAGAGGCTGTCG
25	CTGTTGCCGTTGCGGTATGGCGTGGAAATCAAGTTTTT
26	CTCTCAAACTTAAATTTCATCCGCCGGGCGCGCTCACGCTT
27	GGTGTGTTCGTCATAGTTGAGCTACGTGAACCATC
28	TCGCACTGGGAATGCCAACGGCAGCACGGGTTACCTGTACACT
29	ATTTGCCAAAAATCCCGTAACGGGGTCATTGCAGGGCGGAGGTGT
30	CCGGTGCATGCCGGGGTTTCTAGCCTCCTCTCAG
31	GGTGTGCACTCTGTGGGTCATACCGCGTCGGTGGTGCC
32	GCTGATTAGAAAAAGCCGCACAGGCGGCCTTTAGTGGGTGTACATC
33	ATCCCACGTTCCGGCAGCCTCCCATGCTC
34	CAGCAACCGCAACCAGAGCACAACGTCAAGGATAG
35	GGCCCAGATCCCGTCCGTGGCCAGCATTTTCACGTGCTGCGG
36	ACCCTGCTGGTCTGGACAAACATCCCTGC
37	TGAGAGGCGTTTACCAGTCTGTGCTTAAATCGGAACCC
38	TCTCCGGATAGGTCACGTACGTACAGCGCCATAGGTAAACA
39	CGGATCACGGAAAAGCCGGACCGAGGTGCCGTAAA

40	ATCGGCGGCGGGTTTTCCCAGTCACGATCGTTGGGCGAAACAG
41	TAGATGGGACGACGACAGTAGGATAACCTCACCGGTGTGGAGCCG
42	GACATAAGCCAGCAGTCTCGTTAACGGAACAAGT
43	GAATGAAGGGTAAAGTTCCGTTTTTCGTTGTAAAACGA
44	GTGCCGGACTCGGCCTCAGGAAGATCGCACTCCAGCTTCTGCCAGT
45	CGGCCAACCAGGCAAGGGAAGGAATGGTG
46	TGGGTAACGCCAGATCGGTGCAGGGGGACCCGGAT
47	TGGGGTTTGTAGATCCTCACGCTGGCAAACGCGGTAAACGAT
48	GCACGCAAGGCGATTGTAGAGACATAGAC
49	ACATTACGTCCTGTAGCCACAGAAATTGACGGGGAAAG
50	AACCCTGTGTAGGTAAAGTTCGCGTCTGGCCTGAGCGCCAT
51	GTAATGGTGGGAACGCCAGCTGAGCCCCCGATTTCTCCTTCACTCCT CGTTGC GCGTGGGTGGCGGAACTTAA
52	CAAAAATCGGCAAATATTTAAATTGTAAGGTAACCGTTTGACC
53	AAAGGGTATGATATTCAACCTTAACCAATAGGAACATGTTAAATC
54	TTGAGGGGCGCATCGCTGCGCTTCGCTATTCAGG
55	TTCAGCTTTCCGGCACTCGCCATTCAACGTTAATATTT
56	CTACAAATTGTTCTAGCTGATAAATTAATGCCGGAGAACAAATCAC
57	TGTTAACTATCAGGTCGATGAAAAATTCA
58	AAGATTGTATAAGTAATCGTATGATAATGCTTTAG
59	TAAAGGGGCGAAGGGCCTCAACTGTTAGCGCCATCGCTTCTG
60	GAAGGAGA TAGAGCAGCCCCAAAAAACAAACGGAATGTG
61	GCCTTTTGTGTAATACTTTCGCGAGGGGAAGAAAGCGA
62	AAACTCTTGCTCCTTTTGAAACATTATGACCCCACAATCGG
63	GAGTAATCATATATTATGTACAACGTGGCGAGA
64	TTGTACCACTAGTAGTAGCATTAACATGAGCCGGAGAATGCCT
65	AGGTCATTAATGCTGTAGCTAGAGCATAAAGCTAAGTATTAAGCA
66	CAAGGGTAGCTATTTTGAGAGTCTGCCAATAAATCATA
67	CCATATATCCAACATGTTTTAAATATGCAACTAAAGTACTTAATTG
68	CAGGCAAGTTGATTCATTAGATAAATAAG
69	CAATTCTACTAAATTTCGCAATTTGGGGTGGAAGC
70	CCGGCGCCCGGTAAACTAGAGAGAATCATTGCCTTGAGAGAT
71	AAGGAACTGAAAAGGTGCATTTAAAATTTCA
72	TAATTCTTAAGACTTCAAAATCGTCCAAGTGTAGCGGT
73	TCTACGTTACAGGTAGAAAAGAGGAAGCCCGAAGAGGCATC

74	CCTTTAACAACAGGAGCTATACGGGCGCTAGGG
75	AAAAAGAAGTGCTTTAAACAGTTCAGATAGATGGCTTAGAGTA
76	CATCAGTATACATAACGCCAGAAGCAAAGCGGATTAGCCCTGACT
77	CTGAATATTTTGCGGATTTAGATAACCTGTTTGA
78	TTTACGGTGTCTGGAAGCGAACGAGAAACGAGAATGAC
79	ACCAGACCAAAAGGAATTACGAGGCATAGTAAGAGCCAATTCAACT
80	CATAAACGATAAAAATTGCCAGTTAGATT
81	ATCCCCCTCAAAGGGGTAATACTGCGGATAAAAAA
82	AAGGAGTTTTCAATGGTCATTTGACCCCAATTCTGTTTCATT
83	CGCTGGATAAATATTCATTTCAGGAGAGCTT
84	CTCATTCTTTATGCGATTTCTTGACCGCCGCGCTTAAT
85	AAAACGATCTTTGACCCCCATTGTGAATTACCAACCATTTC
86	AACATTATTAATAAATAGCGTGCGCGTAACCAC
87	AACTTTACGAACGTAACAAAGCTGCTCTTTAGGAATACGGAAC
88	ATTATACCATCGCCTGATAAGCTTGAGATGGTTTAACGAACGAGT
89	AATGCAGTGAGATTTTGCAAAGTTTAGACTATTC
90	ACAACACTATCATAACGCGAGAGGCATTCAGTGAATAA
91	CGCAGACGGATTGTGTCGAAATCCGCGACCTGCTCCACAACGGAGA
92	GGCTTGTCAATCATAAGATGAAATCAGCG
93	ATTACCCAAATCGTGTACAGAGAGTAATTAAACCT
94	CACGCTCCAATAGTAAAATAGAAGTTCCAAAATACCTCGTTT
95	CACACCAAGAACCGGATGGAACGAAATACCA
96	AAAATAAAGGAAGTTTCCACCACGCGGTTGCTTTGACG
97	AAGTTTTTTCTGTATGGGAGACTTTTTCATGATAGTGCTTT
98	AACACTCAAAGAGGTGACCTTCTACAGGGCGCG
99	GAGGACTTATTGCAGGGAGTTAAAGGCAAGAAACAAACACTAA
100	GCTAAACAGGAACAACTAAAGCAACGGCTACAGAGACAGCATCGG
101	TTTGTATCAAGCGCCTTTGAACATAGGCTGATTC
102	AGATGTTACTTAGCCGGAACTGACCCGCTTTTGCGGGA
103	CTCCAAATAGGAATTGCGAATAATAATTTTTTCACGTCGGAGTGAG
104	TCGTCAGAGCCTTTAAATTTCTAAATTTT
105	GGTCGCTGAGGCAACAGCTTGCCATCGCTTATCTA
106	TACTATATAACCGATATGCCAAAAGCGTAAT
107	GCGCCGCATCAACCAGGCGAGAGGACAGGGAACCGAACGAGG
108	CACAGAGAACTACAACGCCTATCACTTAGAATCAGAGC

109	GTTCCAAGGAGTGTACTGCGTCACCAGTACAACGCAGTAAC
110	AATGAATTGTCGTCACAATGATATAACGTGCTT
111	ACTGAGTGGCCTCAGAACCGCCACCCTGCAACAGTTTTTAGTA
112	AAGTTTTTAAACAGTTAATGAGGAACCCATGTACCGCAGGGATAG
113	AGGCTGAATCCCCCTGCCTATTTCGGAACCTATTATCAGTAACAGT
114	TTTTGAAAATCTCCAAGGTTTATCACAGAACCGCCACC
115	CTCAGACTCCTCAAGACCAGGCTTGTAAT
116	TAGTACCGCCACATAAGTGCCATAGGTGTGTTACC
117	AGCACGCAACAAATACCGACGAGGTGATTGTATCAAAAAAGG
118	TCCTCGCGTACTCAGGACGTTTCCACAGCCC
119	AGCCAGATAAACAAATAAAACCGGAAAGGGATTTTAGA
120	ATTAAAATTTGGGAATTAGCCTTGATATTCACGGTTAGGTC
121	ATGATACGTAAGCGAGTATAGTAAACAGGAGGC
122	AGACGATTAAGAACCGCCACCCTCAGACGTCAGTGCCCTTTTG
123	AGCAAAAAAACGTCACCAATACAGGAGGTTGAGGCGAGAGCCGCC
124	GCCCGTAAACGGGGGGGTTTTGGGTTGATACAGA
125	ACTCTGAAACATGAAATAGGATTAGGCCACCACCCTCA
126	CCTTTAGTGGAAACCATCGATAGCAGCACCGTAATCGACATTAGCA
127	GAGCCGTCAGACTGTCCCTTATTGGAGCC
128	GCCGCCACCCTCGCGTTTGCCAGCCACCTCTATTC
129	GGGAGCCCCGGAGTCGAGAGCTCAGTAGAAGGATGTATTAAG
130	CGATTAACCGCCTCCCTTATCATACAATGGA
131	AGGGAGCGAGGGCGACATTGACTCCAGAAGTGTTTTTA
132	TAAAAATGAACACCCTGAGCGCCAAAGACAAATCTTCATAT
133	TTGAGCCGGTGAATTCACCGGCGGTACGCCAGA
134	GGTTTACCGGAAAATACATACATAAAGATTAGCACCAACCGAC
135	GTCAGAGCAAGAATTGAGTTAATCAATAGAAAATTACATAAGTTT
136	AGGCCGGTCACCAGTTTCGGTCATAATCAATATG
137	CGAGTAGCGACAGAATTTCATCGGCGTGGCAACATATA
138	CCGAAGCACAAGCCCAATAATAAGAGCAAGAAACAAGACAGAGAGA
139	AAAGAATTTTTAAGAAGGAAACCAACAAA
140	TTAGCAAACGTAAGGAAACGCTGATTAACATAACA
141	CAGGAAAACCAGATCTTTTCATAGCCAGCGCGTTCAAGTTTG
142	ATCCTGTTATTACGCAGAATATCACGGAAGG
143	AATGAATAGATTTTTTGTTGAAGCCAGAGTCTGTCCAT

144	TAAGTCTTTACGAGCATGTCCAAATAAGAAACGAATATTAT
145	AATTAACCAGGGAAAAAAGAAGTGAGGCCACCGCCTCTGAGATCTCA CAAGGA CTACCGGCACTTAAGTTCCG
146	TTATCCCCTACCCAGCTACAATTTTATGAGAGCGCTACGGGAG
147	ACCAATCCGGGTATTAAACCAAAATAAACAGCCATATGCCTAATT
148	TAACCCAGGTAATTACAAAGTACGGAATACTTAG
149	CATGAAATAGCAATAGCAGATAGCCCCTGAATCTTACC
150	GGAATCAACAAGTACCGCACTCATCGAGAACAAGCATCTTATCATT
151	AACGCTACCGCGCCCCGGTATTAATAGAA
152	TTGCTATTTTGCAAGAACGCGAGGTTTTTAATAGA
153	TAATCACTGGCAAATAATATACCAGAAAAGTAAGCTATCTTA
154	CTCAGAGG AGTAAATTAAATCAAGACCGCGCATAATAGC
155	CTAATGATGACAATAAACAAGTATATACTTCTTTGATT
156	GTCTGATGGGTTATATAATCTGTCCAGACGACAATTAAAGG
157	ATCCTAACTGAACACCCGACTAATTAACCGTTG
158	TAAAGTATATTGAGAATCGCCATATTTATGGCTGTCTTATCCC
159	TGTAAATGAGAAAACTTTTTTATAAAGTACCGACACCGAGCCAGT
160	TTAGCCGTTTTTATTTGCAAATCAGAACAACGCCAACA
161	AAATACCAACAAATATATTTTAGTTAATTTCATCTTACCAAGACAA
162	TGTAATCCGTGTGATTCATAATATCTATA
163	AGTAGGGCTTAACTAGAAAAATCTTACCACCATAG
164	CACGCATGCGGGAGGCGTTGCTTATCAATAGCAATCATCGTA
165	TAGCAAAAGCCAACGCTCTAGAAAACAGAAC
166	AGAAGATAATAGCTTAGATAAACAAAGAAGAACTCAAA
167	ATTGTTAGAACCTACCATTTGAAAACATAGCGACATTTCCC
168	CTTAGGTGAGACTAGCGTTATAACATCACTTGC
169	AATTATTTTTTCAGGTTTAACGCTATTAATTAATTCGCTTGCTTC
170	TTAGAATTTGAATTACCTTTTTTAATGAGCAAATCCACTCCGG
171	AGAACGCGCTGATGAATAAACTAGTATCATTTTA
172	TACTGACCTAAATTTACGTTAAATAGAAACAGTACATA
173	AACGGATGTCGTCAGATGAATATACAGTAACAGTACTAAAGAAATT
174	AATCAAGCCTGATTGCGAATTACCATCAA
175	ACAATTTCATTTCATTTCAATATCAAGATATCCTG
176	AGTAATACAAATGCCTGTTACCGGAAAAATAAGGATGGTTTG
177	CTGAGTAATTAATTACAATCCTTTTGTCAAT

178	TATCAGTGAATTATCATCAAATAGAAGAACAATATTAC
179	AAAACAGCAGAAGATAAAACCAGAAGGAGCGGAAAAGGAAC
180	AAGGGTTTGGATTATGATGAAGCCTTGCTGGTA
181	AAAGAAATAAGACTTTACAAACAATTCCAAAAACAGATAATGG
182	GGTGAGGGCTGAGAGCCAGCACATTATCATTTTGCTAATTTTAAA
183	GCGTAGATGCACGTAAATCGCGCAAAAGAAAGGA
184	ATCTTTTACATCGGGAACCAAGTTAGACAACTCGTATT
185	CCCTCAATAAGCAAATGAAAAATCTAAAGCATCACCAACTGCAACA
186	AAATCCAATATCTGGGGAAGGTCCACAGA
187	TTTAGAAGTATTTCTAAAATAAGCCGTCTAGCCCT
188	CTATCGACAAACTACCTGAGCAGAGGCTTTGAATGAAACAAT
189	ATATCCTAATACATTTGGATACTTCATGATG
190	TTCACCGAGTGAGATAAATCATCCAGTTTGTCAT
191	CATCAATGAGAAAGGCAAACACATGTCAATGCAT
192	AATAGAAAACTTTCTTGCTTTTAGTTGCGCGGTT
193	CCAAGAAAATAATCATAGAAGTTAGCGAACCAAC

staple strand containing the complemental sequence for ODN1	
194	TGCCAGCTGCATTAATGTTTGAATGAAATTATCTACCACAACTCAC
195	GGCTGGTAATGGGTAAATCCAGTCGAAATTATCTACCACAACTCAC
196	TTTCTCCGTGGTGAAGGCCGGGTCAAAATTATCTACCACAACTCAC
197	AGCGAGTAACAACCCGTGGAATTTGAAATTATCTACCACAACTCAC
198	ACGCAAGGATAAAAATTTTTCATCAAAATTATCTACCACAACTCAC
199	CAAAGCGAACCAGACCGCGGGAGAAAAATTATCTACCACAACTCAC
200	GTCAGGACGTTGGGAAGTCGCGTTTAAATTATCTACCACAACTCAC
201	GCCACTACGAAGGCACCAGAACTGGAAATTATCTACCACAACTCAC
202	TCATAGTTAGCGTAACGAAACGGGTAAATTATCTACCACAACTCAC
203	AAGCGCAGTCTCTGAATTAGCATTCAAATTATCTACCACAACTCAC
204	TAAATATTGACGGAAATCTCATTAAAAATTATCTACCACAACTCAC
205	AGCCTTTACAGAGAGAAACCGATTGAAATTATCTACCACAACTCAC
206	GCGCCTGTTTATCAACAACGTCAAAAAATTATCTACCACAACTCAC
207	AGTGAATTTATCAAAATATGTTCAGAAATTATCTACCACAACTCAC
208	GCAATTCATCAATATAAAGACGCTGAAATTATCTACCACAACTCAC
209	TAATGCGCGAACTGATATTCCTGATAAATTATCTACCACAACTCAC

staple strand containing the complemental sequence for ODN2	
210	TTCTGGCCTGTCACACGACCAGTACTTCACACCACACTCCATCTA
211	GTAAAGCCGCCAACATACGAGCCCTTCACACCACACTCCATCTA
212	CCAGCATCGCCGCAACCAGCTTACCTTCACACCACACTCCATCTA
213	CCACGGGAAAGTGCCAAGCTTTCACTTCACACCACACTCCATCTA
214	AGCTCATTGGAATTCGCATTAAATCTTCACACCACACTCCATCTA
215	ATAAAGCCACAGGCAAAGAATTAGCTTCACACCACACTCCATCTA
216	ATTATAGTGATCAAAAATCAGGTCCTTCACACCACACTCCATCTA
217	AGTAAATTGGCCCTGACGAGAAACCTTCACACCACACTCCATCTA
218	AACGAGGGAGCCCTCAGCAGCGAACTTCACACCACACTCCATCTA
219	CAAGCCCAGAGCCACCACCCTCATCTTCACACCACACTCCATCTA
220	GCCAGCATCGCCACCAGAACCACCCTTCACACCACACTCCATCTA
221	ATTTTGTCCCACGCAAAGACACCACTTCACACCACACTCCATCTA
222	TGCCAGTTTTAACGAGCGTCTTTCCTTCACACCACACTCCATCTA
223	AATAAGAGGATTAGGCAGAGGCATCTTCACACCACACTCCATCTA
224	TGTAAATCTCTATATGTGAGTGAACTTCACACCACACTCCATCTA
225	AGTTTGAGTCTTTGCCCGAACGTTCTTCACACCACACTCCATCTA

biotin modified staple strands<br>(In the case of br-RS-3, the following staple strands were used instead of 190-193.)	
biotin modified staple strand 1	biotin-TTTTTTCACCGAGTGAGATAAATCATCCAGTTTGTCAT
biotin modified staple strand 2	biotin-TTTTCATCAATGAGAAAGGCAAACACATGTCAATGCAT
biotin modified staple strand 3	biotin-TTTTAATAGAAAACTTTCTTGCTTTTAGTTGCGCGGTT
biotin modified staple strand 4	biotin-TTTTCCAAGAAAATAATCATAGAAGTTAGCGAACCAAC
